# Evidence-Based Management of Box Jellyfish Stings

**DOI:** 10.1093/milmed/usaf278

**Published:** 2025-09-16

**Authors:** Angel A Yanagihara, Catherine F T Uyehara, Raechel Kadler, Suzanna N Del Rio, Kikiana Hurwitz, Christie L Wilcox, Amanda L Alimboyoguen, Noel Saguil, Matthew C Reed, Jason Barnhill, Breanna Morrison

**Affiliations:** Pacific Biosciences Research Center, School of Ocean and Earth Science and Technology, University of Hawaii at Manoa, Honolulu, HI 96822, United States; Department of Tropical Medicine, Medical Microbiology and Pharmacology, John A. Burns School of Medicine, University of Hawaii at Manoa, Honolulu, HI 96822, United States; Department of Clinical Investigation, Tripler Army Medical Center, Honolulu, HI 96859, United States; Department of Tropical Medicine, Medical Microbiology and Pharmacology, John A. Burns School of Medicine, University of Hawaii at Manoa, Honolulu, HI 96822, United States; Uniformed Services University School of Medicine, Bethesda, MD 20814, United States; Pacific Biosciences Research Center, School of Ocean and Earth Science and Technology, University of Hawaii at Manoa, Honolulu, HI 96822, United States; Pacific Biosciences Research Center, School of Ocean and Earth Science and Technology, University of Hawaii at Manoa, Honolulu, HI 96822, United States; Department of Math and Sciences, Kapiolani Community College, University of Hawaii, Honolulu, HI 96816, United States; Center for Engineering Research and Technology, Polytechnic University of the Philippines, Manila, 1016, Philippines; Department of Quantitative Health Sciences, John A. Burns School of Medicine, University of Hawaii at Manoa, Honolulu, HI 96813, United States; Department of Cell and Molecular Biology, John A. Burns School of Medicine, University of Hawaii at Manoa, Honolulu, HI 96813, United States; Department of Quantitative Health Sciences, John A. Burns School of Medicine, University of Hawaii at Manoa, Honolulu, HI 96813, United States

## Abstract

**Introduction:**

Rapidly acting and highly effective management approaches are critically needed for potentially life-threatening and career-ending stings by box jellyfish (phylum Cnidaria, class Cubozoa) among underwater-operation warfighters working in austere environments. Cubozoan envenomation results in venom load- and time-dependent complex sequelae, including acute-phase hemolysis, cardiorespiratory collapse, hypovolemic shock, and death. Despite previously published studies demonstrating the failure of various generally advised, lay first-aid approaches (including fresh-water rinsing, ice-pack application, and skin scraping) to inhibit box jellyfish venom-induced hemolysis and tissue damage *in vitro*, ineffective and even deleterious management practices persist. In this report, we compared the efficacy of generally used first-aid measures and recently developed copper gluconate (CuGluc)-containing formulations in halting venom-associated tissue damage using a variety of assay systems, including an *in vivo* anesthetized piglet model.

**Materials and Methods:**

The comparative efficacy of common first-aid approaches, including vinegar dousing, hot- and cold-pack applications, gasoline, topical 2-hydroxypropyl-β-cyclodextrin (HPβCD), and novel therapeutics, including CuGluc-containing formulations, was assessed using a variety of platforms, including *in vitro* hemolytic assays, live-tentacle sting tissue-model assays on blood agar or freshly excised porcine skin, and an *in vivo* piglet model.

**Results:**

Sequential topical application of CuGluc-containing formulations (StingNoMore Spray followed by StingNoMore Cream) surpassed all other management approaches in reducing sting-induced hemolysis and tissue damage in all *in vitro* and *in vivo* assay platforms. To a lesser extent, vinegar dousing of the sting site, followed by application of heat (42–45 °C) by hot pack for 45 minutes, also directly and irreversibly inhibited venom activity. Saltwater rinse and ice pack were totally ineffective and led to more tissue damage than the untreated sting itself.

**Conclusions:**

Compared to all other tested first-aid approaches, CuGluc-containing topical spray and cream formulations resulted in far less cubozoan venom-associated tissue damage and represents the most effective method to manage box jellyfish stings.

## INTRODUCTION

Envenomation by jellyfish (phylum Cnidaria, subphylum Medusozoa) can traumatize derma when thousands of stinging cell (cnidocyte) organelles (cnidae) packing each linear centimeter of tentacles ballistically fire. Upon discharge, penetrant cnidae (nematocysts) eject an eversible hollow tubule to inject venom. Across the phylum Cnidaria, nematocysts vary in structure, perforating capacity, and the specific activity of conserved venom components (porins, lipolytic and proteolytic enzymes, and small molecules).^1–9^ Although most stings by Scyphozoa (radially symmetrical jellyfish) and Hydrozoa (e.g., *Physalia*, Hydroids) result in only mild pain and inflammation, stings by Cubozoa (cuboidal bell structure, “box jellyfish”) account for the majority of medically significant and life-threatening sequelae in humans.^2^^,^^3^^,^^8^^,^^9^ Certain cubozoan nematocysts discharge with a break-away arrowhead-like tip, followed by eversion of a spine-covered hollow tubule with axial drill-like rotation, capable of penetrating the carapace of prey and human skin.^4^^,^^5^ Hollow tubules release venom up to 2 mm deep.^6^ Tentacle contact results in thousands of perforating venom-filled wounds per linear centimeter.

**Figure 1.**
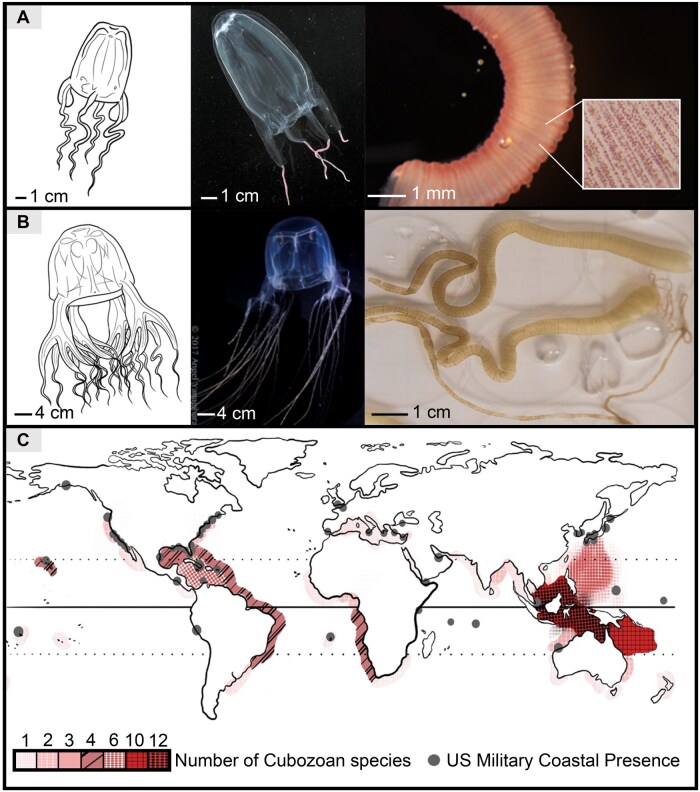
Cubozoan Morphology and Distribution. (A) Morphology of Order Carybdeidae, including *Alatina alata*, and excised tentacles. (B) Morphology of Order Chirodropidae, including *Chironex fleckeri*, and excised tentacles. (C) Distribution of Cubozoan species and overlapping U.S. Military Coastal Presence.

Class Cubozoa comprises 2 taxonomic orders: Carybdeida with 1-3 tentacles emanating from the pedalia at each corner of the cuboidal bell base; and Chirodropida with 4-15 tentacles per pedalia (**Figure 1A** and **B**). Chirodropid mastigophores are the largest and most deeply penetrant nematocysts.^6^^,^^7^ Further, cubozoan venom porins exhibit toxicity orders of magnitude higher than scyphozoan venom porins.^2^^,^^3^^,^^8^ New insights suggest that chirodropid stings can result in venom porin-driven acute hyperkalemia with pulseless electrical activity leading to death within minutes.^8^^,^^9^ Further, porin-driven degranulation of immune cells and platelets after some carybdeid stings correlates with the 4- to 48-hour long sequelae with a concomitant cytokine storm and catecholamine surge, referred to as “Irukandji syndrome” and respiratory effects because of histamine overload.^9^^,^^10^ Symptomatology of Irukandji syndrome includes delayed onset of protracted headache, backache, myalgia, chest pain, abdominal pain, nausea, emesis, diaphoresis, anxiety, an acute hypertensive phase with tachycardia followed by a hypotensive crash with bradycardia, and pulmonary edema with respiratory insufficiency and mortality risk by cerebral hemorrhage.^9^^,^^10^

Underwater operation warfighters and support personnel in certain tropical and subtropical regions (∼30°N to 30°S) face cubozoan envenomation risk from over 40 box jellyfish species (**Figure 1**C). Respective habitats, feeding, and reproductive behaviors are order- and species-specific. Generally, chirodropids reach adulthood at the warmest period of the year (before seasonal monsoon rainfall-driven low salinity mass die-off events) in shallow mangrove estuaries.^11^^,^^12^ This ecology correlates with reports of severe injury and deaths from stings (principally among children, within 50 m of the beach) near mangrove estuaries and their outflows in the Philippines, Indonesia, Australia, Papua New Guinea, Malaysia, and during night spawning aggregations in Thailand.^11^^,^^12^ By contrast, most carybdeids present year-round pelagic threats with one species (*Alatina alata*) exhibiting lunar-cycle synchronized coastal spawning aggregations.^13^ Many pelagic carybdeid species feed near the ocean surface during nocturnal invertebrate vertical migrations.^14^ For these reasons, systematic field ecological surveys are critical to assess potential risks and hazards for a given locality.

Trainees at the Special Forces Underwater Operations (SFUWO) School in Fleming Key, Key West, Florida, have suffered serious envenomation by several species of night-feeding cubozoans^15^ in near-shore seagrass habitats. In the past 15 years, more than 12 well-documented sting cases were recorded among SFUWO trainees who displayed cardiovascular symptoms consistent with Irukandji syndrome.^15^^,^^16^ Severe stings (4 in March 2009; 5 in May–June 2010, >6 in 2013) represent substantial impediments to mission completion and force health protection (*personnel communications, SFUWO dive medical officers*). The critical need to develop rapidly acting and highly effective management of cubozoan stings prompted this work.

Translational limitations of certain *in vitro* experimental models may explain the conflicting cnidarian sting^17–22^ first-aid assessments comprising this extant literature. Unfortunately, these contradictory recommendations are echoed in current online medical practitioner guidelines (i.e., eMedicine, Cleveland Clinic, Seattle Children’s, Healthdirect, UCLA Health, Merck Manual, Australian Red Cross). Unlike live-tentacle induced hemolysis time-course blood-agar-based methods, solution based “sting” models comprising only excised tentacles with added test solutions assess cnidae rupture, but do not directly assess venom activity, which is the driver of tissue injury in real-world settings.^17^^,^^23^ Furthermore, secondary cytolytic activity recovered from lyophilized vinegar-rinse solution after battery-stimulated tentacle discharge^24^ yielded activities orders of magnitude below authentic stings (µg/mL versus ng/mL or pg/mL). Unfortunately, some models have led researchers to claim, solely by extrapolation, that well-established approaches with no retrospective proof of harm, such as vinegar, could be dangerous.^24^ More appropriate approaches to evaluate rinse solutions require live reporter cells such as intact erythrocytes in blood agar, where venom activity can be continuously and directly measured.^17^ During an authentic sting, triggered sensory-hair cell bundles elicit nematocyst discharge. The victim’s skin is perforated during the discharge of thousands of nematocysts, but not all. Some remain intact, undischarged, and adherent to the skin surface. Although no longer innervated by sensory hair cells and far less reactive, discharge can still be triggered by osmotic shock (e.g., fresh-water rinse) and other physicochemical triggers (e.g., rubbing).^19^ Application of rinse solutions to these undischarged, isolated nematocysts in a blood agar tissue model or *in vivo* allows for direct evaluation of damaging venom ejection versus cnidae inactivation or innocuous cnidae rupture.

Another reason for contradictory first-aid recommendations is reliance on participant-reported pain scores at short time points to assess “first-aid” effectiveness. Cubozoan stings involve hours-long venom release with complex pathophysiological sequelae. Thus, short-term pain-scores do not necessarily reflect either sting severity or long-term outcomes of “treatment” approaches. This is very clearly illustrated in time-course venom-activity models, in which slow-releasing venom shows increasing tissue damage over an 18-hour test period.^19^ The clinical presentation of stings while sometimes delayed, (e.g., Irukandji syndrome) and involving minimal sting-site pain, can be life-threatening.^25^ Taken together, these complex phenomenologies require a complex suite of experimental models to appropriately assess efficacy of candidate first-aid approaches over time-course studies.

To directly assess potential inhibitors of venom activity, standard red blood cell (RBC) solution-based hemolytic assays were performed over concentration ranges of both venom and potential inhibitors. Similarly, *in vitro* hemolytic blood agar-based assays and *ex vivo* piglet skin models were designed to quantitate live-tentacle envenomation-induced hemolysis or tissue damage in time-course sequelae alone or after the application of “first-aid” approaches.^17^ Based upon the highly conserved nature of venom components among cubozoans, representative chirodropids and carbydeids were chosen for first-aid evaluation assays.^1^ Hemolytic activity was first evaluated in an (1) RBC-solution assay. Then, live, spontaneously stinging tentacles were applied to (2) live erythrocytes in blood agar plates, (3) freshly-harvested porcine skin, and (4) anesthetized piglets. Dose- and time-course effects after exposure to venom or to live- tentacle stings were compared with outcomes after common first-aid approaches, including saltwater rinsing followed by ice packs, vinegar rinse, heat packs, gasoline (commonly used in the Philippines) and newly described approaches: 2-hydroxypropyl-β-cyclodextrin (HPβCD),^26^ and copper gluconate (CuGluc)^9^^,^^17^^,^^19^^,^^27^ alone and in topical formulations.

## MATERIALS AND METHODS

### Animal Collections

*Alatina alata* (family Carybdidae) were collected along Waikiki Beach in Honolulu, HI, USA, as described previously,^5^^,^  ^8^^,^  ^28^ and *Chironex fleckeri* (family Chirodropidae) were collected in Mapoon, Cape York, Queensland, Australia, as well as in Lucena, and Marabut, Samar, Philippines.

### Vertebrate Animal Research Ethics Statement

The Institutional Animal Care and Use Committee of Tripler Army Medical Center approved all experimental procedures on live piglets (Protocol No. 17A10), in accordance with guidelines of the Department of Health and Human Services Public Health Service Policy and US Department of Agriculture Animal Welfare Act.

### Venom Hemolytic Activity Assay

The hemolytic activity assay was modified from previously published protocols.^8^^,^^28^ Briefly, whole blood (University of Hawaii Institutional Biosafety Committee, Protocol B22-100618) was washed with phosphate-buffered saline (PBS) at low-speed centrifugation (500*g*, 20 minutes, 4 °C). In each well of a 96-well V-bottom plate, a 2.5% RBC solution in PBS was incubated with potential inhibitors: 5 mM HPβCD or 0.5 mM CuGluc for 5 minutes, followed by the addition of serially diluted *A. alata* venom. Alternatively, serially diluted *A. alata* venom was added to the RBC solution, followed by potential inhibitors at 5 minutes for post-treatments. Following incubation for 1 hour at 37.5 °C, the plate was centrifuged (500*g*, 5 minutes), the supernatant was transferred to a flat-bottomed 96-well plate, and absorbance was read at 405 nm (Ultramark EX, Bio-Rad Microplate Imaging System, Bio-Rad Microplate Manager Software, Version 5.2.1). Reference samples included 1% Triton-X for complete hemolysis and 2.5% RBC in PBS for zero hemolysis. The dilution levels (µg/mL) compared were: 375, 150, 9, 0.7, 0.17, 0.04, 0.01, 0.003, 0.0007, 0.0002, 0.00001. The treatments compared were: venom alone, pre- and post-HPβCD and pre- and post-CuGluc. A 2-way analysis of variance (ANOVA) comparing the treatments and different dilution levels was done with a Tukey post hoc test to compare the different conditions to the control condition for significant effects. A one-way ANOVA comparing the normalized values of treatments at a selected venom concentration (0.17 µg/mL) was done with a Sidak’s multiple comparisons test to evaluate significance.

### Tentacle Blood Agar Assay (TBAA)

Functional venom delivery and activity was determined over a 12+-hour time-course using conventional MacConkey 5% sheep’s blood agar plates (e.g., Remel Blood Agar) using *A. alata* or *C. fleckeri* tentacles. Tentacles replete with cnidae were excised and collected into trays using forceps and scissors. For pre-treatments, the blood agar was coated with either 0.5 mM CuGluc in PBS or 5 mM HPβCD in PBS for 10 minutes before the addition of cubozoan tentacles. Tentacles were removed after 5 minutes (*A. alata)* or 3 minutes (*C. fleckeri*) of stinging. For post-sting treatments, tentacles were laid on the agar for the respective stinging-period, followed by addition of the potential inhibitors directly to the blood agar. For heat and ice treatments, hot (45 °C) or cold (0–3 °C) packs were placed over the blood agar for 45 minutes, following the sting (Hot packs, Bent Grass Concepts LLC, Greenwood Village, CO, USA; Cold packs: CVS Instant Cold Pack, CVS Pharmacy Inc., Woonsocket, RI, USA). Plates were incubated at 37.5 °C and images were recorded at time intervals using a dissecting microscope (Olympus model SZX16, Olympus Corporation, Tokyo, Japan or Nikon D800). The hemolytic zone was calculated using ImageJ software.^17^ The time points were: 1, 3, 6, and 12 hours for *A. alata* and an additional time point of 15 hours for *C. fleckeri*. The conditions compared were: no treatment, pre- and post-HPβCD, pre- and post-­CuGluc, hot-pack and cold-pack for *A. alata*. Previous published work demonstrated statistically significant inhibition of *C. fleckeri* tentacle sting-induced hemolysis by CuGluc-­containing spray.^19^ A mixed ANOVA compared the different conditions and time points; a Bonferroni test was used to determine significance.

### Porcine Skin Stinging Assay

Fresh post-mortem piglet skin sections were obtained from commercial butchers in Lucena, Philippines. Skin sections were delaminated from subdermal fat, and sterilized with rapid rinsing (3 minutes, twice) in 10% ethanol, 5% hydrogen peroxide, and 110 mM sodium chloride (NaCl), followed by sterile 0.9% NaCl. Skin sections were set in frames (5.5″ × 6.4″) to keep the skin taut. Chirodropid tentacles were prepared as previously described in *Tentacle Blood Agar Assay*. Piglet skin sections were designated to receive the following test measures following stings from the freshly excised chirodropid tentacles: vinegar, StingNoMore Spray, StingNoMore Spray and Cream, gasoline, or no treatment. Four excised tentacles were placed on each piglet skin for 20 minutes. Test measures were administered to their respective skin grafts, and images were taken with a Nikon D800 over an 18-hour time course to provide qualitative data.

### *In vivo* Live-Tentacle Porcine Model

Six piglets (each weighing approximately 8 kg) were used for the live tentacle sting time-course assay. Piglets were anesthetized in a supine position (for access to the inguinal and axillary areas) and given a background intravenous infusion (normal saline, 0.1 mL/kg/min). Volume infusion was sustained throughout to maintain adequate hydration and central venous pressures as determined at baseline. After an initial 60- to 90-minute period of stabilization after catheterization, pigs were assessed for a 20-minute period to define their baseline.

*A. alata* tentacle preparation followed the same procedure previously described in the *Tentacle Blood Agar Assay* section. Once piglets were assessed at baseline, approximately 20 *A. alata* tentacles were placed at each of three sites. A parafilm layer and 0.9% NaCl pouch was applied atop the tentacles for direct contact of the tentacles with the skin. The tentacles stung for 5 minutes, and test measures were administered 10 minutes post-tentacle removal. For each piglet, 2 of 3 sting sites received a designated test measure for 45 minutes, and 1 did not receive treatment as a control. Test measures evaluated were StingNoMore Spray followed by StingNoMore Cream and 0.9% NaCl followed by an ice-water pack (0–3 °C). The experiment proceeded for 4 hours post-sting with time-point images using a Nikon D800. Postmortem, 8 mm cross sections of the epidermal, dermal and subdermal layers were fixed with formaldehyde, then stained with hematoxylin and eosin for analysis.

## RESULTS

### Venom Hemolytic Activity Assay

**Figure 2.**
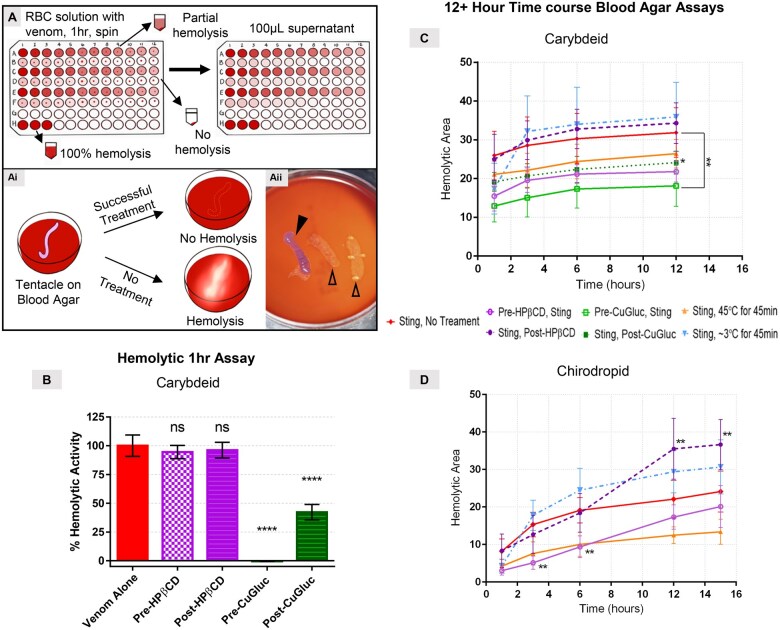
*In vitro* assays: 96-well hemolytic assay and tentacle blood agar assay. (A) Schematic of hemolytic and (Ai) Tentacle Blood Agar Assays (TBAA). (Aii) Representative image of TBAA. Enclosed arrowhead indicates an *Alatina alata* tentacle laid upon blood agar. The empty arrowheads indicate areas of hemolysis following tentacle removal. (B) % Hemolytic activity of pre- and post-treatment of 0.5 mM Copper Gluconate (CuGluc) and 5 mM 2-Hydroxypropyl-β-cyclodextrin (HPβCD) with *A. alata* (0.17 µg/mL) exposed 1% RBC solution at 1 hour. Significance was determined by Sidak’s multiple comparisons test. (*P*-value: **** < .0001, ns = not significant). (C) Time-course hemolysis of *A. alata* tentacle exposed blood agar and (D) *Chironex fleckeri* tentacle exposed blood agar with pre- and post-treatment with 0.5mM CuGluc, 5mM HPβCD, Heat, or Ice. Significance determined by Bonferroni test or unpaired *t*-test (*P*-value: ** < .05, * < .1).

A schematic of a 96-well plate hemolytic assay of RBC solution exposed to venom is shown in **Figure 2A**. The hemolytic activity of *A. alata* venom alone was compared to pre- and post-treatments of 0.5 mM CuGluc and 5 mM HPβCD (**Figure 2B**). At 0.017 µg/mL, the lowest concentration at which venom alone still caused complete hemolysis (a serious sting equivalent dose), both pre- and post-CuGluc significantly reduced hemolytic activity (**Figure 2B**) (*P *< .0001). Both pre- and post-CuGluc were significantly lower than both pre- and post-HPβCD (**Figure S1**). The highest concentration (375 µg/mL) represents ∼100× the lethal dose equivalent based on 1-m tentacle contact for an adult 5 L blood volume. Prophylactic, pre-CuGluc was significantly effective even at a ∼2.5× lethal dose equivalent at 10 µg/mL and inhibited hemolytic activity at a higher venom concentration than all other treatments evaluated.

### Tentacle Blood Agar Assays (TBAA)

Schematic and exemplar images of TBAA are shown in **Figure 2A**. For *A. alata* tentacle-exposed blood agar (**Figure 2C**), pretreatment with 0.5 mM CuGluc significantly reduced hemolytic area across all time points (*P* < .05) compared to control. At a 90% confidence interval, the post-CuGluc treatment resulted in significantly less hemolysis as compared to the control (*P* = .0615) at the 12-hour time point. The hemolytic area following pre- and post-treatment with HPβCD, post-CuGluc treatment, or heat treatment after tentacle exposure was not significantly different from the control at 1 hour. Importantly, although cold pack (0–3 °C) treatment for 45 minutes resulted in a similar hemolytic area to the control at 1 hour, later time points showed accelerating hemolysis, with the highest hemolysis observed at the final time point (12 hours). Application of live *C. fleckeri* tentacles without treatment constituted baseline hemolytic areas over 15 hours (positive control, **Figure 2D**). Post-treatment with HPβCD resulted in significantly more hemolysis at both 12 hours (*P = *.0066) and 15 hours (*P = *.028) compared to the control. Cold-pack (0–3 °C) treatment was not significantly different from the baseline, although the hemolytic area was greater for every time point after 1 hour, following the same trend as *A. alata* tentacle exposure and cold-pack treatment. Pre-HPβCD and heat-pack treatments were consistently lower than the control, but only statistically significant at 3 and 6 hours.

### Porcine Skin Stinging Assay

**Figure 3.**
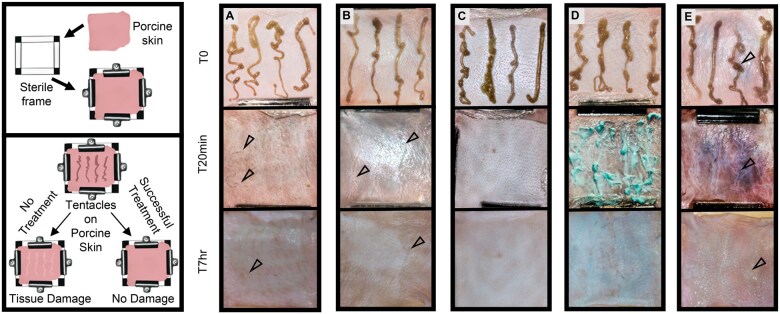
*In vitro* porcine skin assay with freshly excised *Chironex fleckeri* tentacles. *C. fleckeri* tentacles were placed on newly-butchered porcine dermis for 20 minutes, followed by application of varying test measures. (A) Control, no treatment, (B) Vinegar, (C) Sting No More Spray, (D) Sting No More Spray and Cream, (E) Gasoline.

Live *C. fleckeri* tentacles were applied to fresh post-mortem porcine skin (**Figure 3**). Images were taken over a 7-hour time course for qualitative data (**Figure 3A–E**, **Figure S2**). Porcine skins exposed to tentacles and without treatment (i.e., positive control), showed visible wounding with pitting at 7 hours (**Figure 3A**, empty arrowheads). Similarly, post-tentacle exposed vinegar-rinsed skins showed pitting along tentacle contact sites (**Figure 3B**, empty arrowheads). Notably, post-tentacle exposed copper-gluconate containing StingNoMore Spray-treated as well as StingNoMore Spray and Cream-treated skins showed no visible sign of skin pitting or damage (**Figure 3C** and **D**, respectively). By contrast, gasoline rinsing after tentacle exposure caused massive aberration of the skin with visible acute-phase delipidation (**Figure 3E**), dermal liquefaction and tissue distortion (**Figure S2**).

### *In vivo* Porcine Live-Tentacle Sting Model

**Figure 4.**
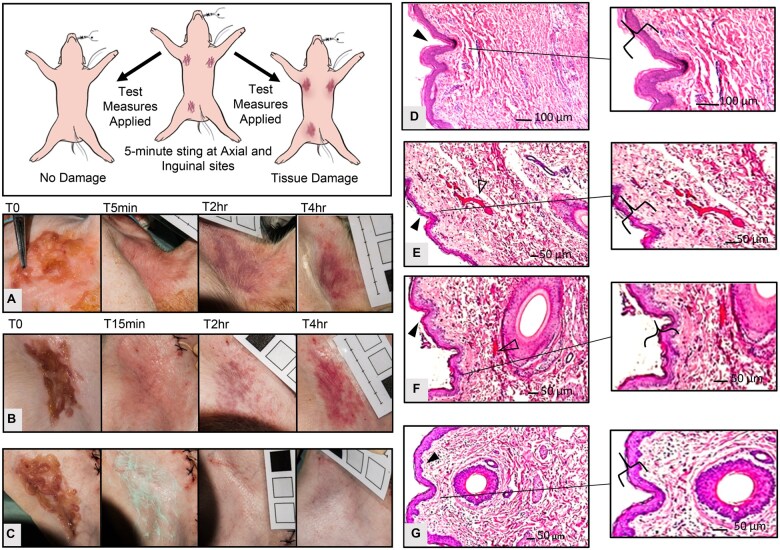
*In vivo* porcine skin assay with freshly excised *Alatina alata* tentacles. (A–C) Tentacles were placed on axial and inguinal sites of anesthetized piglet(s) for 5 minutes, followed by application various test measures. (A) Left axillary site, control (5-minute sting, no treatment). (B) Right inguinal site, saline spray and ice pack (5-minute sting, saline spray to remove tentacles, 10-minute rest, ice pack for 45 minutes). (C) Right inguinal site, StingNoMore Spray and StingNoMore Cream (5-minute sting, StingNoMore spray to remove tentacles, 10 minute rest, StingNoMore Cream for 45 minutes). (D–G) Representative histology (10× magnification). (D) Control, No sting or treatment, (E) Tentacle exposed, no treatment, (F) Tentacle exposed, saline spray and ice pack, and (G) Tentacle exposed, StingNoMore Spray and StingNoMore Cream.

An *in vivo* anesthetized porcine model was used to examine time-course outcomes of various “first-aid” approaches, following a 5-minute sting by *A. alata* tentacles (**Figure 4**). As shown in **Figure 4A–C**, images were taken of sting sites on live piglets to assess tissue damage over time. Tentacle exposure alone (**Figure 4A**) was compared to potential mitigation approaches at 15 minutes post-sting, either a saline rinse followed by 45 minutes of cold-pack (0–3 °C) (**Figure 4B**), or StingNoMore Spray then 45 minutes of StingNoMore Cream (**Figure 4C**). Tissue damage was most evident with saline and cold-pack treatment (**Figure 4B**). StingNoMore Spray followed by StingNoMore Cream had minimal tissue damage in comparison to the control.

### Postmortem Porcine Dermal Histopathology

Skin cross-sections composed of the epidermal, dermal and subdermal layers were analyzed microscopically (**Figure 4D–G**). Tentacle exposed skin (**Figure 4E–G**), show histopathology compared to nontentacle-exposed (i.e., control) skin (**Figure 4D**). Specifically, marked basophil and neutrophil infiltration occurred in the tentacle-exposed skin (**Figure 4E**) as well as with saline and ice-pack treatment (**Figure 4F**). In contrast, tentacle-exposed skin treated with StingNoMore Spray and Cream (**Figure 4G**) did not show marked leukocyte infiltration. There was visible damage to the dermal and epidermal integrity of both untreated tentacle-exposed skin, as well as with saline and ice-pack treatment. Further, hemorrhage and increasingly engorged blood vessels because of congestion was documented in both of these groups (**Figure 4E–F**, empty arrowheads). StingNoMore Spray and Cream-treated tentacle-exposed skin (**Figure 4G**) maintained epidermal and dermal integrity (solid arrowhead) with no hemorrhage. The subdermal zone barrier remained distinct, with minimal neutrophilic infiltration at the subcutaneous layer.

## DISCUSSION

### Rationale and Limitations

The critical need for rigorous, evidence-based management protocols for life threatening cubozoan stings prompted these studies. In addition to previously published *in vitro* approaches,^8^^,^^17^^,^^19^ piglet *in vivo* models were utilized to independently evaluate first aids and to re-evaluate sting-care recommendations based upon extrapolations of narrowly designed single-experimental assay approaches.^24^ This unique study design comprising multiple and independent direct approaches (*in vitro*, *ex vivo*, and *in vivo* models) provides in-depth insight into envenomation pathophysiology and efficacy of various treatment approaches.

The limitations of the complementary approaches include the following. Firstly, although biochemical approaches to recover bioactive venom from tentacle nematocysts demonstrate hemolytic assay activity, the question remains whether these biochemically purified venom preparations^8^^,^^29^ or saline extracts^30–33^ qualitatively and quantitatively recapitulate an authentic sting. Secondly, RBC solution-based end-point assays are time delimited (typically 1 hour). Using live, spontaneous stinging tentacles in multi-hour time-course assays address these limitations. Thus, the blood agar- and *ex vivo* porcine-skin assays utilized in this study allow for a more thorough evaluation of first-aid effects or clinical measures and recapitulate a live sting in using freshly excised live tentacles. However, envenomation-based downstream pathophysiological effects inherent in clinical cases^9^ can only be observed with *in vivo* models. For these reasons, we designed time-course assays of live-tentacle stings in anesthetized piglets both acutely and over a time course to assess first aid in this study to provide the greatest insight into management implications.

### Testing of Extant First-Aid Approaches

Extant first-aid approaches include rinsing with saltwater, vinegar,^17^^,^^19^^,^^24^ or gasoline; hot- or cold-pack application,^18^ or newly described approaches: topical HPβCD,^26^ CuGluc^9^^,^^17^^,^^19^^,^  ^27^ StingNoMore Spray^9^^,^^17^^,^^19^^,^^27^ (containing CuGluc, vinegar, MgSO_4_, and urea), and StingNoMore Cream^9^^,^^17^^,^^19^^,^^27^ (containing CuGluc, calciferol, and urea, all compounded into Lipoderm (PCCA, Houston, TX, USA). Comparable to previous studies,^17^^,^^19^^,^^27^ CuGluc significantly reduced venom-induced hemolysis in both hemolytic assays and TBAA (**Figure 2B, C**). Live tentacles spontaneously discharge when laid on the surface of blood agar; the observation that hemolytic zones continue to increase over 12 hours illustrates an important limitation to solution-based studies and those with shorter time points. Although the hemolytic area was similar for the control and post-HPβCD treatment at earlier time-points, HPβCD resulted in significantly more hemolysis at 12 and 15 hours. By contrast, CuGluc continued to inhibit hemolytic activity throughout the 12-hour incubation period. Thus, time-course data are essential in evaluating first-aid approaches. The data also support using hot-water immersion, or hot packs, after stings, as skin-safe heat application (45 °C) significantly reduced hemolysis in our models (**Figure 2C, D**). For both *A. alata* and *C. fleckeri* tentacle-exposed blood agar followed by ice-pack treatment, hemolytic areas were similar to the control at 1 hour, but surpassed and were consistently higher than the control by 2 hours. Thus, the definitive outcome of various mitigation methods cannot be assessed at arbitrarily short time points, such as 1 hour; a time-course analysis is imperative (**Figure 2C, D**). Although the mechanism of enhanced tissue damage after ice treatment is not entirely clear, it correlates with RBC solution-based assays.^28^ This appears to reflect the thermodynamics of the porin monomer to polymer transition required for functional pore formation^8^: colder temperatures augment self-assembly of the porin monomers into polymeric functional pores, whereas patient-tolerable heat (45 °C) markedly inhibits this necessary self-assembly.^8^ These data demonstrate ice-pack treatment exacerbated tissue destruction and wounding.

Vinegar dousing has been commonly advised as “first aid” to remove adherent tentacles.^17^^,^^34^^,^^35^ Our results show that vinegar prevented additional penetrant cnidae discharge (**Figure 3B**) from the carybdeid and chirodropid species tested, but it did not inhibit venom already injected into the sting site, or the subsequent time course of progressive tissue damage. Thus, vinegar alone is not an authentic “treatment,” but is a prudent first-aid measure to prevent additional stinging by undischarged cnidae left on skin after tentacle contact. However, vinegar did not, as some suggested,^18^ exacerbate or worsen stings. These data do not support the idea that vinegar “can kill” or should not be used.^24^ Instead, our results indicate that medical practitioners should be aware of vinegar’s limitations.

### Development of Novel Therapeutics

Exploratory work over 15 years in search of novel therapeutics, including the rigorous testing of more than 50 candidate venom inhibitors, resulted in the identification of divalent metal gluconates of zinc and copper.^27^ Although effective in inhibiting venom pore-forming protein-based hemolysis and in rescuing mice injected with lethal venom doses,^8^ zinc gluconate (half-maximal inhibitory concentration, IC50, 5 mM) was subsequently found to increase the harmful activity of venom zinc-dependent metalloproteinases. By contrast, CuGluc had greater capacity (IC50, 50 µM) to inhibit venom-induced hemolysis,^27^ without the activation of zinc-dependent matrix metalloproteinases. CuGluc is a safe copper salt that is used in neonatal nutritional support (e.g., Baxter IV Junyelt). Thus, given the lack of evidence of any deleterious effects on venom-induced tissue damage, and its inclusion in FDA monographs as a generally recognized as safe and effective (GRASE) agent for topical administration in over-the-counter (OTC) use within the concentration ranges needed, CuGluc was formulated into a spray and greaseless topical cream for first-aid use in sting victims, in collaboration with the University of Hawaii Daniel K. Inouye College of Pharmacy and in consultation with SFUWO dive medical officers. Highly effective CuGluc-based topical 2-step venom-inhibitor products were developed, comprising a liquid spray formulation (StingNoMore Spray, which also contained urea and vinegar to inactivate adherent stinging cnidae by rinsing) and a rapidly absorbed cream formulation (StingNoMore Cream, compounded with the pharmaceutical grade base Lipoderm).^27^ Topical application of StingNoMore Spray and StingNoMore Cream in both *ex vivo* and *in vivo* porcine models showed minimal or no tissue damage compared to the control and alternative treatment methods (**Figures 3** and **4**).

Examination of first-aid efficacy was performed using an anesthetized piglet model (**Figure 4**). First-aid treatments applied to stings by live *A. alata* tentacles on piglets included StingNoMore Spray followed by StingNoMore Cream, which showed the highest level of inhibition of venom-induced tissue damage (**Figure 4C**). StingNoMore Spray and StingNoMore Cream first-aid products have been publicly available (http://stingnomore.com) and used by SFUWO and other military components since 2016. These are fully compliant with all extant rules and regulations, including GS1 registration.

## CONCLUSIONS

Multiple sting model assays, including live cubozoan tentacle stings in an anesthetized piglet model with postmortem histology, demonstrate the utility of CuGluc-containing topical spray and cream formulations (i.e., StingNoMore Spray and StingNoMore Cream) to stop envenomation-based hemolysis, tissue damage and inflammation, as well as provide topical prophylaxis. To a lesser extent, vinegar rinse followed by 45 °C hot pack treatment for 20–45 minutes reduced tissue damage. By contrast, saline-rinse and ice-pack treatments worsened injuries.

## Supplementary Material

usaf278_Supplementary_Data

## Data Availability

The data that support the findings of this study are available on request from the corresponding author.

## INSTITUTIONAL ANIMAL CARE AND USE COMMITTEE (IACUC)

This study was approved by the Tripler Army Medical Center Institutional Animal Care and Use Committee (IACUC) (Protocol No. 17A10), in accordance with guidelines of the Department of Health and Human Services Public Health Service Policy and US Department of Agriculture Animal Welfare Act.
