# Supplementary material for: Evidence-Based Management of Box Jellyfish Stings
Source: Mil Med. 2025 Sep 16;190(Suppl 2):589–98. doi: 10.1093/milmed/usaf278 (PMC12448702; doi:10.1093/milmed/usaf278)
Supplement: usaf278_Supplementary_Data [file usaf278_supplementary_data.zip › Appendices - Supplemental Figure 1.pptx]

## Slide 1
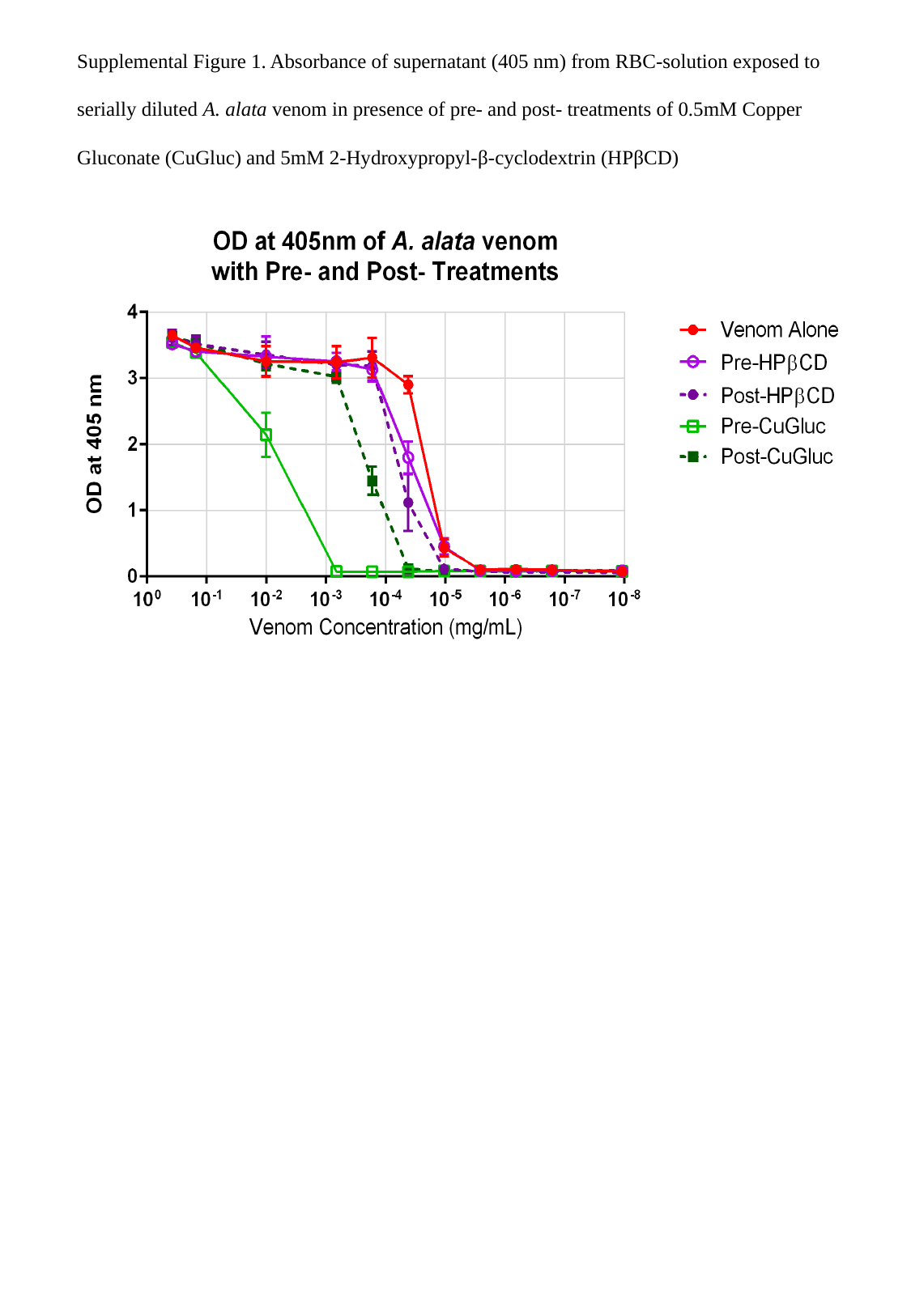

Supplemental Figure 1. Absorbance of supernatant (405 nm) from RBC-solution exposed to serially diluted A. alata venom in presence of pre- and post- treatments of 0.5mM Copper Gluconate (CuGluc) and 5mM 2-Hydroxypropyl-β-cyclodextrin (HPβCD)
