# Supplementary material for: Evidence-Based Management of Box Jellyfish Stings
Source: Mil Med. 2025 Sep 16;190(Suppl 2):589–98. doi: 10.1093/milmed/usaf278 (PMC12448702; doi:10.1093/milmed/usaf278)

Supplemental Figure 2. Raw image of porcine skin at 7 hour timepoint. Treatments of the skin are as follows: Top row from left to right: Vinegar, 2-Hydroxypropyl-β-cyclodextrin (HPβCD) Cream, Control (No treatment). Middle row from left to right: StingNoMore Spray, StingNoMore Cream, StingNoMore Spray & Cream. Bottom row left to right: Sand, Gasoline.

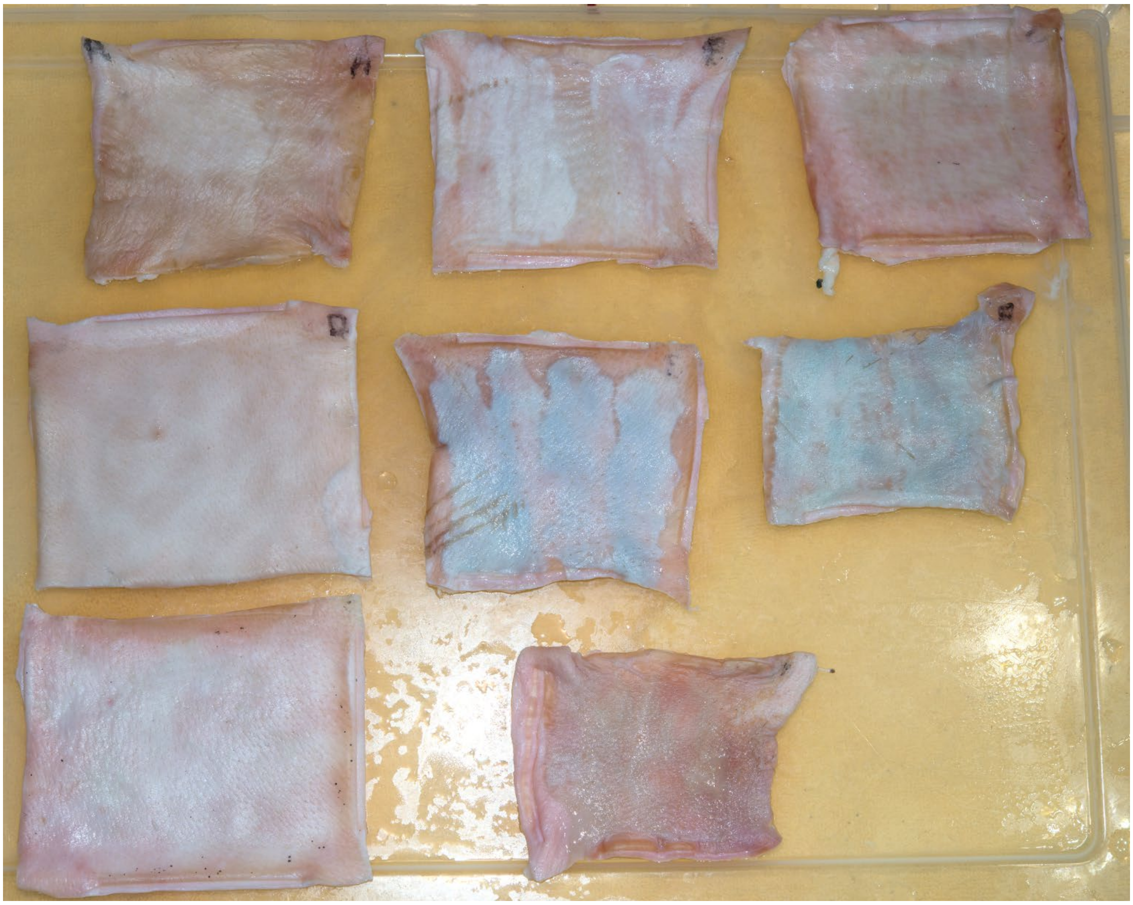

Supplement: usaf278_Supplementary_Data [file usaf278_supplementary_data.zip › Appendices - Supplemental Figure 2.pdf]
